# Extension of Tablo TrEatmeNt Duration (XTEND) study: successful 24 h prolonged therapy with Tablo in critical patients

**DOI:** 10.1186/s12882-022-02968-4

**Published:** 2022-10-21

**Authors:** Tahir Zaman, Kasadi Moore, Jennifer Jellerson, Yaadveer Chahal, Joshua Schumacher, Cynthia Dalessandri-Silva, Michael Aragon

**Affiliations:** 1 Mountain Star Healthcare Network, Salt Lake City, USA; 2 Outset Medical, San Jose, USA

**Keywords:** Prolonged therapy, ICU, COVID, Alarms

## Abstract

**Background:**

The Tablo® Hemodialysis System (Tablo) is an all in one, easy-to-learn device featuring integrated water purification, on demand dialysate production and two-way wireless data transmission and is approved for use in the acute, chronic, and home settings. Prior reports have demonstrated Tablo’s ability to achieve clinical goals, seamlessly integrate into hospitals and reduce cost across a wide range of treatment times. Extension of the Tablo cartridge to 24 h allows prolonged therapy and even greater flexibility for prescribers in the acute setting. The objective is to report on the first ever experience with Tablo prolonged therapy between 12 and 24 h in critically ill patients treated at a single-center ICU.

**Methods:**

Nursing staff were trained during a single training session on Tablo prolonged therapy. After a run-in period of five treatments, Tablo data were collected via real-time transmission to a cloud-based, HIPAA compliant platform and reviewed by site staff. Dialysis treatment delivery, clinically significant alarms, and clotting events were recorded. Sub-group analysis between COVID-19 positive and negative patients were reported.

**Results:**

One hundred (100) consecutive Tablo prolonged treatments had a median prescribed treatment time of 24 h and a median achieved treatment time of 21.3 h. Median cartridge usage was 1.3 per treatment. The dialysis treatment time was delivered in 91% of treatments, with 6% ending early due to an alarm, and 3% ending due to clotting.

Clinically significant alarms occurred at a median rate of 0.5 per treatment hour with a resolution time of 18 s. Median blood pump stoppage time related to these alarms was 2.3 min per treatment. Blood pump stoppage time was higher in the COVID-19 subgroup when compared to the non-COVID-19 subgroup.

**Conclusion:**

Tablo successfully achieves prescribed treatment time with minimal therapy interruptions from alarms or cartridge changes. This data demonstrates the effectiveness of Tablo in achieving personalization of treatments necessary for unstable patients and enabling successful delivery of extended therapy with minimal clotting. Tablo’s prolonged therapy meets the needs of critically patients, including COVID-19 positive patients, requiring renal replacement therapy for greater than 12 h.

## Introduction

Renal replacement therapy (RRT) is often required to treat critically ill patients with acute kidney injury (AKI) or end-stage kidney disease (ESKD) in the intensive care unit (ICU) [1, 2]. AKI occurs in up to half of all patients admitted to the ICU, and of these patients, 4-13% require RRT [1, 3]. While there are various RRT modalities available, including continuous, prolonged, and intermittent, meta-analyses have demonstrated minimal differences in clinical outcomes between these modalities [4]. However, ICU clinicians typically favor treatments of greater than 12 h for hemodynamically unstable patients [5–7], as they allow for more gradual fluid removal and solute clearance over the course of the treatments [5, 8].

The same severe illness that leads to the need for a prolonged therapy often results in organ failure beyond AKI; further complicating the treatment regimen with other critical lifesaving devices, such as mechanical ventilation and extracorporeal membrane oxygenation (ECMO) therapy, that can lead to interdevice interaction and competition for vascular access [9, 10]. Prolonged therapy is frequently administered concurrently with ECMO, either independently or integrated into the circuit, to mitigate hemodynamic instability and fluid imbalance [11] presenting a highly complex and labor-intensive treatment regimen.

The acuity of these patients, combined with the increased treatment time and slower blood flow rate, adds complexity to the effective delivery of prolonged therapies. The most common complication is associated with circuit clotting or clogging. Clotting in the filter or extracorporeal circuit may be exacerbated by compromised or inadequate vascular access [12]. Poor catheter function may trigger arterial and venous pressure alarms which stagnate extracorporeal blood flow, leading to increased blood pump stoppage that exacerbate clotting events [5, 13]. During the pandemic, the risk of clotting or clogging the circuit during prolonged therapies was further increased in COVID-19 positive patients with AKI due to the prothrombotic state induced by the disease process [14]. Clotting and clogging of the extracorporeal circuit can have significant impact on the ability to achieve ultrafiltration and metabolic clearance targets due to treatment interruptions. In addition to the risk of failing to achieve clinical goals, these interruptions drive up the cost of care due to increased supply utilization from repeated system set up [15], and create additional work for an already highly burdened critical care nursing staff [16].

Taken together and compounded by the staffing shortages that have resulted from the COVID-19 pandemic, there is a critical need for simple, flexible RRT technology that can deliver effective therapy with minimal treatment interruption. The Tablo® Hemodialysis System (“Tablo” or “Tablo System”) is a novel FDA approved all-in-one system capable of purifying water and producing dialysate on demand in any environment, from the ICU to home. It consists of a console and a single use, disposable extracorporeal circuit (the Tablo Cartridge) for hemodialysis. Tablo is compatible with most commercially available high-flux dialyzers and can perform hemodialysis, isolated ultrafiltration, or sequential, mixed-mode treatments without the need for external water purification devices or sterile bags of dialysate.

Tablo can deliver dialysis therapy across a wide range of blood and dialysate flow rates on a single cartridge up to 24 h. The aim of the Extension of Tablo Treatment Duration (“XTEND”) study was to provide the first ever report of treatment experience, cartridge utilization, and clotting events in critically ill patients prescribed treatments between 12 and 24 h using the Tablo System.

## Materials and methods

### Study design and patient eligibility

An observational, retrospective, post-market study was conducted at St. Mark’s Hospital (Salt Lake City, UT, USA) over a 9-month period between January 2021 and September 2021 to report on the first 100 consecutive treatments performed beyond 12 h on the Tablo system. The study was reviewed and approved by the Advarra Institutional Review Board (IRB) as the IRB of record (Pro00049220). In accordance with IRB guidance, a waiver of informed consent was granted, as the standard of care encompasses the study intervention. Hospital staff underwent standard training performed by Outset Medical, Inc. (“Outset”) personnel on prescribing, setup and management of the Tablo console.

After an initial run-in period at the facility of five Tablo treatments greater than 12 h, all consecutive treatments were screened for eligibility. All patients who were hospitalized in the ICU with ESKD or AKI and requiring dialysis were screened for eligibility. Inclusion criteria consisted of patients who weighed 34 kgs or greater and were prescribed RRT for greater than 12 h utilizing Tablo.

### Procedures and data collection

Demographic information, sequential organ failure assessment (SOFA) scores [17, 18], and clinical data was obtained from each patient’s electronic medical records. Prescribed and actual Tablo treatment parameters were collected through real-time transmission to the TabloDash online resource (a cloud-based, HIPAA compliant platform hosted by Outset). All other data including treatment duration, blood pump stoppage time, alarm time to resolution, and total number of cartridges used per treatment were obtained from device sensor data or from Tablo’s automated electronic flowsheets and reports. The delivered dose of RRT was calculated by total effluent volume (L), per hour of actual treatment time achieved, per patient unit weight (kg).

Clinically significant alarms were defined as high or low venous or arterial pressure, low systolic blood pressure, air in the venous bloodline and dialyzer blood leak alarms. Any additional alarm(s) that led the device to direct to end treatment to ensure patient safety were categorized as “Additional Safety Alarms” (e.g., dialysate conductivity alarm).

Treatment outcome determination was performed by hospital nursing staff and were grouped into one of three categories: 1) dialysis treatment time delivered; 2) ended due to an alarm; or 3) ended due to clotting. Dialysis treatment time delivered was defined as those treatments that achieved at least 90% of the prescribed treatment time or delivery of treatments that achieved less than 90% of the prescribed time but were deemed sufficient by clinicians at the site such that re-initiation of treatment was not clinically indicated. Treatments ending due to an alarm included both user and device-directed end treatments where additional setup and same day re-initiation of dialysis treatment was required. Treatments that ended due to clotting were defined as those ended early with documented visual confirmation of clotting within the extracorporeal circuit by the treating nurse.

Descriptive statistics were used to describe study results. For continuous variables, median, mean, standard deviation (SD), and range values were obtained. For categorical variables, percentage proportions were calculated by dividing the number of events by the total number of patients or treatments.

## Results

### Patient characteristics and baseline data

A total of 100 consecutive prolonged Tablo treatments in 33 critically ill patients were analyzed and included in the analysis. Twenty-six COVID-19 negative patients received 74 treatments (“non-COVID-19”). Seven COVID-19 positive patients received 13 treatments (“COVID-19”), and one COVID-19 positive patient undergoing concurrent ECMO therapy (“COVID-19 + ECMO”) received 13 treatments.

Given the impact of COVID-19 positivity on clotting, data was analyzed as a comparison of patients testing positive or negative for COVID-19. The COVID-19 + ECMO patient was analyzed separately as an outlier due to the potential skew of treatment results associated with the delivery of dialysis and ECMO within a single circuit. Data for this patient is presented at the end of the results section.

The median patient age was 55 years with a mean weight of 109 kg and SOFA score of 13. Most patients were female (56%), white (69%), and had a non-cuffed temporary catheter placed for RRT access (94%). Aggregate baseline demographics for the 32 non-COVID-19 and COVID-19 patients are presented in Table 1.

**Table 1:** Baseline characteristics stratified by positive or negative COVID-19 status (*n* = number of patients)

Patient Demographics	Total	Non-COVID-19	COVID-19
	(*n* = 32)	(*n* = 26^a^)	(*n* = 7^a^)
*Age* (yr)			
18 – 34	19% (6)	23% (6)	-
35 – 64	53% (17)	46% (12)	86% (6)
≥ 65	28% (9)	31% (8)	14% (1)
*Admission Weight* (kg)^b^			
< 89	35% (11)	40% (10)	17% (1)
90 – 119	42% (13)	40% (10)	50% (3)
> 120	23% (7)	20% (5)	33% (2)
*Sex*			
Female	56% (18)	62% (16)	43% (3)
Male	44% (14)	38% (10)	57% (4)
*Ethnicity/Race*			
White	69% (22)	73% (19)	57% (4)
Hispanic	9% (3)	8% (2)	14% (1)
Polynesian	6% (2)	8% (2)	-
Native American	6% (2)	8% (2)	-
Asian	3% (1)	-	14% (1)
Other	3% (1)	4% (1)	-
Unknown	3% (1)	-	14% (1)
*Access Placement*			
Non-Tunneled Catheter	94% (30)	92% (24)	100% (7)
Tunneled Catheter	3% (1)	4% (1)	-
Combination^c^	3% (1)	4% (1)	-
*SOFA Score*^b^			
≤ 12	31% (10)	32% (8)	43% (3)
> 12	66% (21)	68% (17)	57% (4)

^a^Patients are not mutually exclusive since treatments were performed both prior and post their COVID-19 positive status

^b^Admission weight and SOFA score for one non-COVID-19 patient was unavailable

^c^One patient initiated their hospital stay with a non-cuffed tunneled catheter then transitioned to a permanent catheter

One patient was admitted twice during the period of observation and is represented in both the COVID-19 and non-COVID-19 groups. The initial hospital admission required Tablo therapy of greater than 12 h in the ICU for non-COVID-19 related illness during which the patient tested negative for the virus. The patient was readmitted into the ICU five months later, during which the patient tested positive for COVID-19, and again required Tablo therapy of greater than 12 h.

The mean prescribed dialysis session length (DSL) per treatment was 24 h. All treatments used a high flux dialyzer and dialysate calcium bath of 2.5 mEq/L. The majority were prescribed a 4 mEq/L potassium bath (83%), a median sodium of 140 mEq/L (135–145) and bicarbonate of 35 mEq/L (30–40). Median prescribed dialysate fluid temperature was 36.0° C (35.0–38.0). A continuous infusion of heparin per site protocol was utilized in 46% of the treatments.

### Treatment parameters

A total of 1,855 h of prolonged RRT on Tablo were analyzed. The mean dialysate flow rate (DFR) was 101 ± 60 mL/min, with a blood flow rate (BFR) of 176 ± 40 mL/min. Treatment parameters and heparin usage are presented in Table 2. A total of 99% of treatments achieved a delivered RRT dose of > 20 mL/kg/hr per treatment, with a mean total ultrafiltration (UF) rate of 3.2 ± 3.1 mL/kg/hr.

**Table 2:** Treatment parameters stratified by positive or negative COVID-19 status (*n* = number of treatments)

Treatment Parameters	Total	Non-COVID-19	COVID-19
	(*n* = 87)	(*n* = 74)	(*n* = 13)
*Blood Flow Rate* (mL/min)			
< 150	8% (7)	9% (7)	-
150 – 200	87% (76)	85% (63)	100% (13)
> 200	5% (4)	5% (4)	-
*Dialysate Flow Rate* (mL/min)			
< 100	37% (32)	27% (20)	92% (12)
100 – 150	53% (46)	65% (44)	-
> 150	10% (9)	6% (4)	8% (1)
*Cartridge Usage*			
1 Cartridge	79% (69)	81% (60)	69% (9)
2 Cartridges	13% (11)	11% (8)	23% (3)
3 Cartridges	8% (7)	8% (6)	8% (1)
*Heparin Drip Used*	46% (40)	45% (33)	54% (7)

**Fig. 1:**
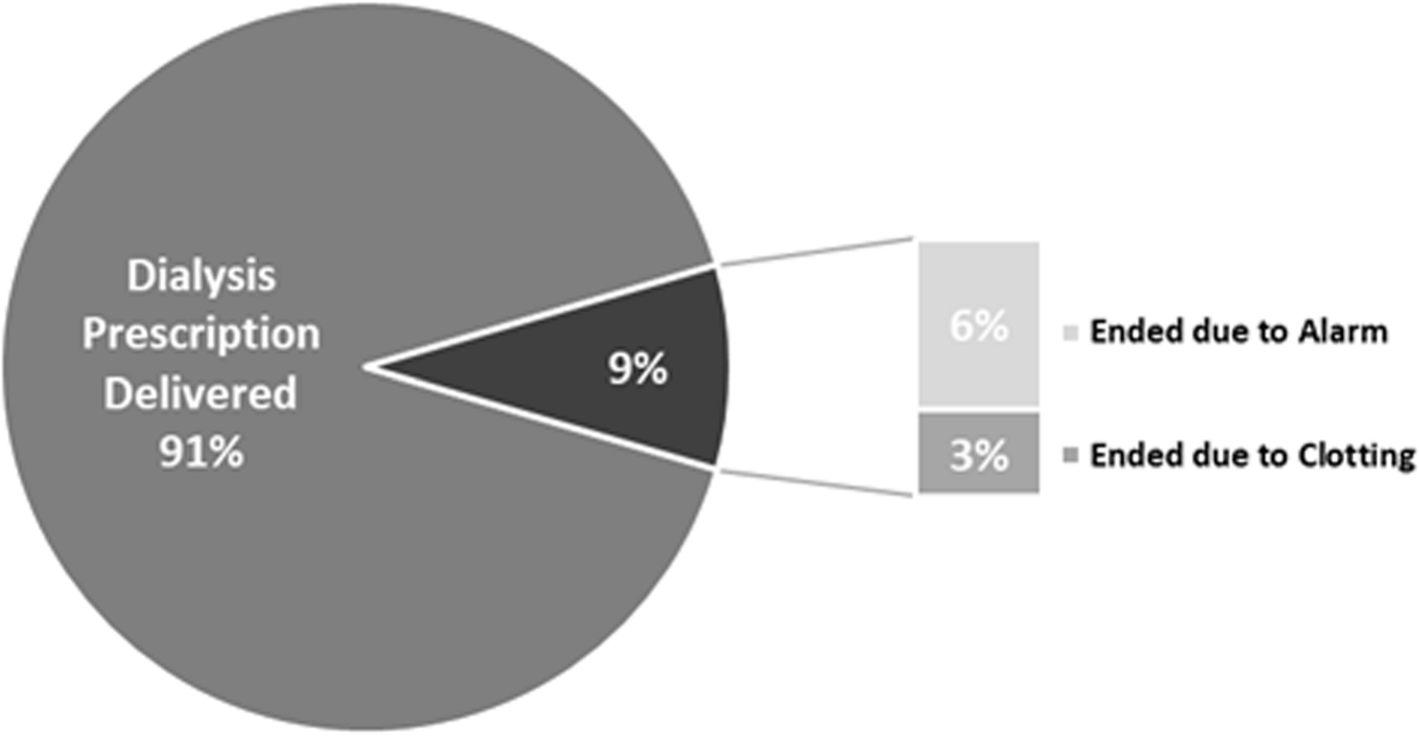
Individual Treatment Outcomes (*n* = 87)

**Fig. 2:**
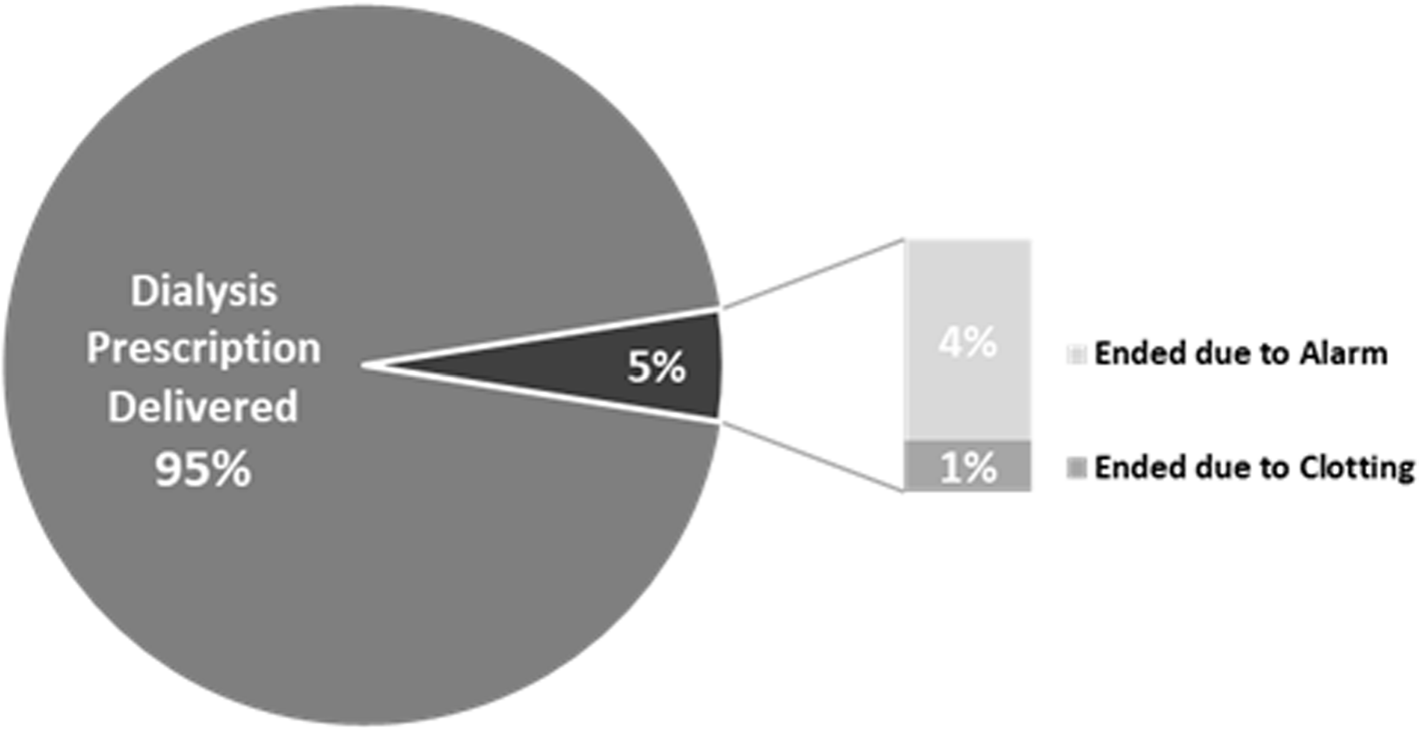
Individual Treatment Outcomes for Non-COVID-19 Treatments (*n* = 74)

**Fig. 3:**
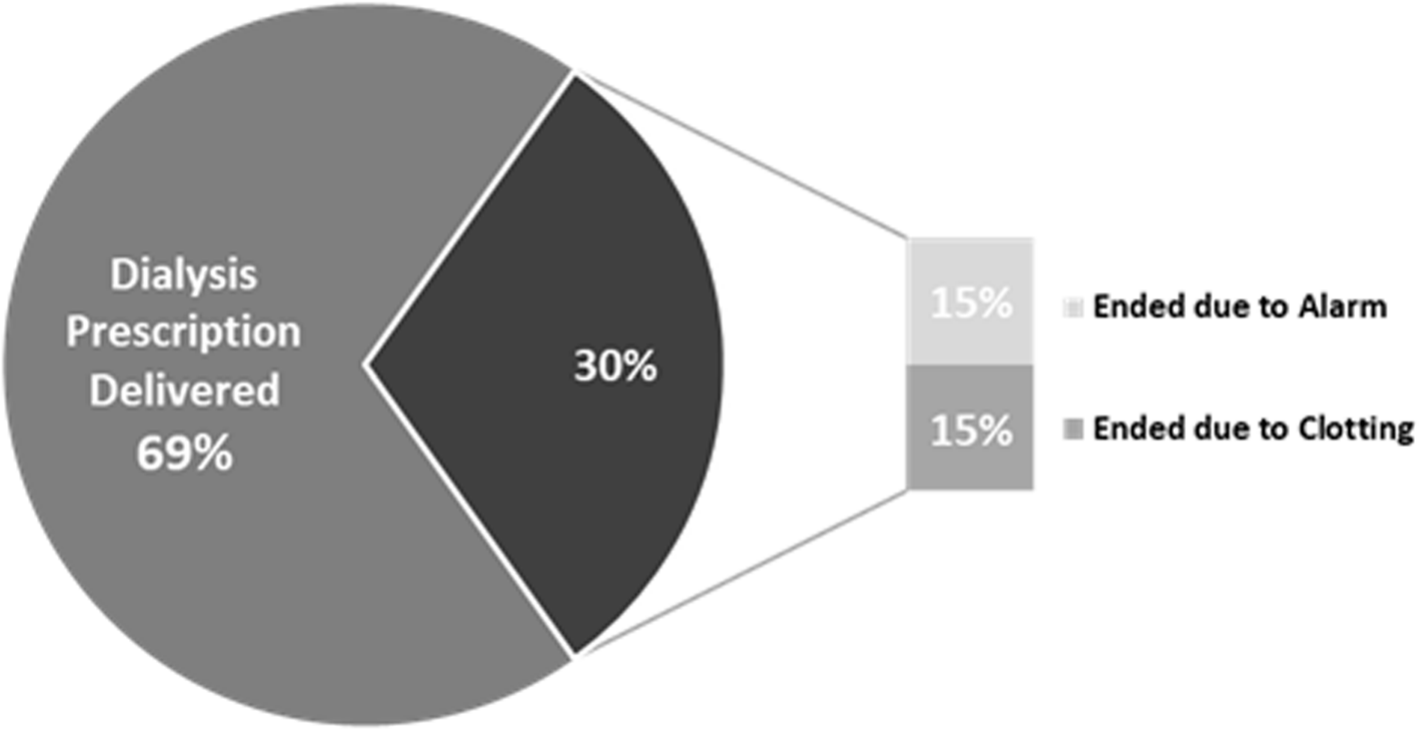
Individual Treatment Outcomes for COVID-19 Treatments (*n* = 13)

The dialysis treatment time was delivered in 91% of treatments, with 6% ending early due to an alarm, and 3% ending due to clotting. Of the five treatments (6%) ending due to an alarm, two were related to arterial pressure alarms that resulted in a user-directed end of treatment and the remaining three involved device-directed alarms that ended treatment to prioritize patient safety. These were due to air in the venous bloodline (1), incorrect dialysate conductivity reading (1), and saline infusion delay (1). See Figs. 1, 2 and 3 for treatment outcomes.

Of the three treatments (3%) that ended due to clotting, one occurred in a non-COVID-19 patient that was on a heparin drip, with the remaining two treatments occurring in COVID-19 patients who were not on a heparin drip.

### Clinically significant alarms

A total of 985 clinically significant alarms were observed, resulting in a median overall rate of 0.4 alarms per treatment hour. Median rates of clinically significant alarms in the non-COVID-19 and the COVID-19groups were 0.4 (0–3.4) and 0.5 (0–3.0) per treatment hour, respectively.

The median time to alarm resolution by site staff in the ICU was 14 s. Alarms for non-COVID-19 treatments were resolved at a median time of 13 (3–116) seconds, while alarms for COVID-19 treatments were resolved in 21 (3–64) seconds. Alarms were related to venous pressure (59%), arterial pressure (40%), and additional safety alarms (< 1%). Median blood pump stoppage time related to clinically significant alarms was 2.3 min per treatment. Blood pump stoppage time was higher in the COVID-19 subgroup with a median of 11.4 (0.1–22.5) minutes when compared to the non-COVID-19 with a median of 2.2 (0–20.7) minutes per treatment. Mean values for alarm data are presented in Table 3.

**Table 3:** Clinically significant alarm data stratified by positive or negative COVID-19 status (*n* = number of treatments)

Alarm Parameter (Mean ± SD)	Total	Non-COVID-19	COVID-19
	(*n* = 87)	(*n* = 74)	(*n* = 13)
*Alarm Rate per Hour*	0.5 ± 0.6	0.5 ± 0.6	0.7 ± 0.9
*Alarm Resolution Time* (seconds)	18 ± 18	16 ± 17	26 ± 20
*Blood Pump Stoppage Time* (minutes)	4.5 ± 5.2	3.7 ± 4.2	8.8 ± 7.7

### Cartridge usage

The average cartridge usage per treatment was 1.3 ± 0.6 with 79% (69 treatments) requiring the use of a single cartridge (refer to Table 2). Increased cartridge utilization was associated with a higher rate of clinically significant alarms per hour of 1.7 ± 0.9 compared to treatments utilizing one cartridge (0.4 ± 0.4). Of note, the mean cartridge usage in the non-COVID-19 (1.3 ± 0.6) and COVID-19 (1.4 ± 0.7) groups was similar.

### COVID-19 + ECMO patient

One COVID-19 + patient requiring RRT received concurrent ECMO therapy. Thirteen Tablo treatments were performed during the patient’s hospital admission via a temporary dialysis catheter, with an admission weight of 124 kg and SOFA score of 13. The dialysis treatment time was delivered in 77% (10/13) of all treatments, with two treatments ending due to arterial pressure alarms and one treatment ending due to additional safety alarm related to air in venous bloodline.

The alarm rate for the COVID-19 + ECMO treatments occurred at a median rate of 2.0 (0.6–8.1), with a median alarm resolution time of 9 (4–14) seconds. The median blood pump stoppage time was 15 (4–46) minutes. The median UF rate was 5.2 ± 1.0 mL/kg/hr. Median cartridge usage for the COVID-19 + ECMO treatments was 1.8 ± 1.5, with three treatments requiring greater than three cartridges to deliver the intended therapy.

## Discussion

The use of the Tablo Hemodialysis System for up to 24 h in this critically ill population resulted in high clinical treatment success, low cartridge utilization, minimal clotting, short average blood pump stoppage times and low rates of clinically significant alarms which were resolved quickly; irrespective of COVID status. These factors all likely contributed to the dialysis treatment time being delivered in 91% of treatments, with 99% of treatments achieving or exceeding target effluent rates of > 20 ml/kg/hr [5].

Over the course of 100 treatments, only three treatments were discontinued due to visible clotting. Two of the three occurred in COVID-19 patients, which is consistent with the increased risk of circuit clotting reported in the literature [19]. There remained a single clotting event in a non-COVID-19 patient on a heparin drip.

The rate of clinically significant alarms per 24-h treatment period was low and was accompanied by rapid alarm resolution. The mean blood pump stoppage time observed in this study (4.5 ± 5.2 min per treatment), trended lower than previous reports from a conventional RRT device (6.7 ± 9.5 min) [20]. The increase in blood pump stoppage times in the COVID-19 subgroup can be attributed to the higher number of total alarms per treatment and higher mean time to alarm resolution. The alarm resolution time in this subgroup was likely due to the need for ICU staff to don PPE prior to entry into the treatment room.

As previously mentioned, the COVID-19 + ECMO patient was excluded from the broader analysis due to the increased complexity of managing both therapies within a single extracorporeal circuit. Increased alarm rates were noted during the initial hours of therapy and were due to the adjustment of stopcock positioning to optimize the combined ECMO/Dialysis extracorporeal circuit (data not shown). This increased blood pump stoppage time may have contributed to the high cartridge usage that was observed with this patient. Additionally, while data concerning the incidence of clotting in RRT requiring COVID-19 + ECMO patients are scarce, the existing literature supports that these patients are at an elevated risk of experiencing clotting events [11, 21] and may also have contributed to the results observed.

### Strengths

This is the first study to provide detailed data concerning the expanded capabilities that Tablo can now deliver on a single cartridge for up to 24 h. The study evaluated Tablo’s performance in a real-world setting, which is particularly valuable as it demonstrates Tablo can successfully deliver adequate renal replacement therapy to critically ill patients, including those with COVID-19 and undergoing ECMO therapy. As COVID-19 surges are likely to continue well into the future [22], these data further support that Tablo therapy prescribed for greater than 12 h can perform treatments with minimal interruptions and decrease the overall burden on nursing staff associated with cartridge changes, complex alarm troubleshooting, and clotted extracorporeal circuits.

### Future directions

The minimal clotting events and low cartridge usage that were observed are incredibly encouraging, especially in a vulnerable population at increased risk of clotting due to COVID-19 infection [23, 24]. Although not utilized as part of this study, Tablo has the ability to deliver a scheduled saline flush that can automate the delivery of up to 300 mL of saline every 60 min during treatment. While further evidence is needed, prior reports on the use of saline flushes in outpatient intermittent hemodialysis suggests the benefit of reduced clotting during treatment [25, 26]. Additional studies in acute care settings are required to evaluate the effectiveness this feature may have on clotting outcomes and help fill evidence gaps related to anticoagulation strategies in patients requiring prolonged therapies [27]_._

While this study was not set up to determine any specific cost benefits associated with the use of Tablo in the ICU, future trials comparing Tablo’s cartridge lifespan to that of filters used in conventional RRT devices are warranted, as higher filter replacement represents a substantial financial burden to hospital systems [15]. This review did not examine circuit lifespan as it relates to COVID-19 patients requiring prolonged therapies. A recent study on this population reported a median filter life of only 6.5 h [14]_,_ further highlighting the positive results presented here.

### Limitations

While the XTEND trial is the first report of Tablo utilization for greater than 12 h in critically ill patients with key insights into the effectiveness in the acute care setting, there are several limitations to the study. This observational study was not sufficiently powered and involved a small sample size without a control group. Additionally, the treatment success outcomes were subjective and based on assessments made by the clinical expertise of hospital staff. A randomized clinical trial examining multiple, well defined endpoints is needed to appropriately determine the efficacy of Tablo compared to conventional RRT devices. While patient SOFA scores were recorded and assisted in determining acuity, principal diagnosis or primary reason for hospital admission were not included and therefore limit the broader application of these results to a specific AKI population requiring RRT (e.g., sepsis, post-surgical, trauma patients). This also applies to patients admitted to the ICU primarily for COVID-19 complications, as it is possible patients included in this study may have been positive for SARS-CoV-2 upon admission, but not actively experiencing acute COVID viral illness. Furthermore, since the study was conducted at a single center, we are unable to generalize these results. While evidence-based clinical practice guidelines have been published regarding the prescription and delivery of RRT, there is wide variation in treatment implementation amongst institutions [28], which necessitates a larger, multi-center trial to adequately expand upon the findings reported here.

Given the above limitations, the results of the XTEND trial support that Tablo is effective in the treatment of critically ill patients in the ICU who require prolonged therapies, including COVID-19 positive patients. The results presented suggest that Tablo’s low alarm rates, rapid alarm resolution, minimal blood pump stoppage time and low cartridge usage per 24 h can reduce the overall cost and nursing staff burden traditionally associated with the provision of extended dialytic therapy in the ICU.

## Data Availability

The datasets generated and/or analyzed during the current study are not publicly available due to the study sponsor’s liability of competitive exposure. They are available from the corresponding author on reasonable request.
